# Preparation of Activated Carbon-Based Solid Sulfonic Acid and Its Catalytic Performance in Biodiesel Preparation

**DOI:** 10.3389/fchem.2022.944398

**Published:** 2022-06-21

**Authors:** Yuanzheng Pi, Wenzhu Liu, Jiani Wang, Guanmin Peng, Dabo Jiang, Ruike Guo, Dulin Yin

**Affiliations:** ^1^ College of Chemistry and Materials Engineering, Huaihua University, Huaihua, China; ^2^ National and Local Joint Engineering Laboratory for New Petro-Chemical Materials and Fine Utilization of Resources, Hunan Normal University, Changsha, China

**Keywords:** halogenated benzene, activated carbon, sulfonic acid catalyst, palmitic acid, biodiesel

## Abstract

With activated carbon as raw material, AC-Ph-SO_3_H was prepared after oxidation with nitric acid, modification with halogenated benzene and sulfonation with concentrated sulfuric acid. After modified by 10% bromobenzene with toluene as a solvent for 5 h, followed sulfonation with concentrated sulfuric acid at 150°C, the -SO_3_H content of prepared AC-Ph-SO_3_H was 0.64 mmol/g. Acid content test, infrared spectroscopy and Raman spectroscopy detection proved that the surface of AC-Ph-SO_3_H was successfully grafted with -SO_3_H group. When used as a catalyst for the methylation of palmitate acid, the catalytic performance of AC-Ph-SO_3_H was explored. When the reaction time was 6 h, the amount of catalyst acid accounted for 2.5 wt% of palmitic acid, and the molar ratio of methanol/palmitic acid was 40, the esterification rate of palmitic acid was 95.2% and the yield of methyl palmitate was 94.2%, which was much better than those of its precursors AC, AC-O, and AC-Ph (both about 4.5%). AC-Ph-SO_3_H exhibited certain stability in the esterification reaction system and the conversion rate of palmitic acid was still above 80% after three reuses.

## 1 Introduction

Fossil energy plays an extremely important role in the national economy and people’s livelihood, and is a strong driving force for human progress and social development (Darda et al., 2019; Antar et al., 2021). With the rapid development of the global industry, the consumption of oil resources is increasing day by day, resulting in a decrease in oil reserves and serious environmental problems. More and more countries have begun to pay attention to new energy sources that can replace fossil energy. Biodiesel is considered to be an effective substitute for fossil energy with the advantages of high safety in use, excellent environmental protection properties, good ignition performance and good lubricity (Gebremariam and Marchetti, 2018a; Singh et al., 2020). Homogeneous acid catalysts such as sulfuric acid, nitric acid, and phosphoric acid are often used in the traditional production process of biodiesel (Su, 2013; Gebremariam and Marchetti, 2018b). However, homogeneous acid catalysts have some unavoidable defects, such as strong protonic acid catalysts that are easy to corrode production equipment during the reaction process, difficult to separate and recycle when mixed with products after the reaction, and generate waste acid and pollute the environment, etc. (Wancura et al., 2020) Therefore, in the pursuit of green and sustainable development, the application of homogeneous acid catalysts is becoming less and less popular. Heterogeneous catalysts, which can perfectly avoid the above problems, have attracted more and more attention of researchers (Jayakumar et al., 2021; Esmi et al., 2022). However, many existing heterogeneous catalysts also have some shortcomings, such as high catalyst preparation cost, low catalytic activity, slightly poor reuse performance, long time and high temperature for catalytic reaction, etc. (Hsiao et al., 2020; Mukhtar et al., 2022) Therefore, the development and preparation of highly active, low-cost, and multi-functional heterogeneous catalysts used in catalyzing the synthesis of biodiesel, has far-reaching significance for realizing the effective replacement of fossil energy and promoting social development.

For the advantages of high acid density, good thermodynamic stability, excellent surface hydrophobicity, high chemical stability and other characteristics, carbon-based solid acids have gradually been developed into popular catalysts that attract the interest of many researchers (Vakros, 2018; Tang and Niu, 2019; Tobío-Pérez et al., 2022). In addition, carbon-based solid acid catalysts can be prepared using inexpensive and renewable biomass and its derivatives as raw materials (Rocha et al., 2019; Wilk et al., 2020), and the required production cost is low. Rocha (Rocha et al., 2019) prepared sulfonated activated carbon from corn cobs and used it as catalyst for biodiesel production using microwave-assisted transesterification of soybean oil with ethanol, and the yield of product was 88.7%. While there are some problems such as poor stability and easy shedding of acidic functional groups due to the leaching of–SO_3_H groups or small molecular fragment on surface structure of amorphous carbon (Ma et al., 2020; Yusuff et al., 2022). Tang (Tang and Niu, 2019) synthesized a series of carbon-based solid acid catalysts from bamboo, which showed a strong acid density of 1.28 mmol g^−1^ and excellent catalytic performance in esterification reaction, but the biodiesel yield declines to 83.7% after fourth reused cycle of the catalyst. Ma et al. (2020) produced microporous lignin-derived carbon-based solid acid with lignin as a carbon precursor, and used it in the esterification of oleic acid with methanol. The conversion of oleic acid reached 92.3% but reduced to 72.9% after recycling for five times. Using skeleton-stabilized activated carbon materials to prepare solid acids to avoid the shedding of surface functional groups is an effective way to prepare carbon-based solid acids. But due to the stable structure of the activated carbon surface, there are few sulfonable functional groups on the surface, and it is difficult to be grafted with strong acid functional groups directly with sulfonation. Therefore, the development of new methods to introduce sulfonable functional groups on the surface of activated carbon is of great significance for the preparation of activated carbon-based solid acids.

In this research, in view of the fact that there are basically no sulfonated sites on the surface of activated carbon (AC) materials, oxidation with nitric acid was used to destroyed the surface structure of activated carbon and lead to generate a certain amount of oxygen-containing functional groups, which can be coupled with halobenzenes to create sulfonated sites on the surface. Then activated carbon-based solid sulfonic acid (denoted as AC-Ph-SO_3_H) was further prepared by using concentrated sulfuric acid as sulfonating agent. The effect of modification conditions on the content of sulfonic acid groups on AC surface was investigated, and its stability in different solvents was also investigated. The catalytic performance of the prepared AC-Ph-SO_3_H in the preparation of biodiesel by methyl esterification of palmitic acid was investigated and discussed.

## 2 Experimental

### 2.1 Materials

Activated carbon (AR, Xilong Chemical Co., Ltd., China); concentrated hydrochloric acid (AR, Chongqing Chuandong Chemical Co., Ltd., China); concentrated sulfuric acid (98%) (AR, Chengdu Jinshan Chemical Reagent Co., Ltd., China); concentrated nitric acid (AR, Guangdong Guanghua Technology Co., Ltd.,China). Other reagents were all of analytical grade and purchased from Sinopharm Group Chemical Reagent Co., Ltd., China.

### 2.2 Preparation of AC-Ph-SO_3_H

With AC as raw material, AC-Ph-SO_3_H was prepared by sulfonation with concentrated sulfuric acid after oxidizing with nitric acid and modified by halogenated benzene. The general process of preparation was as follows: 2 g activated carbon and 20 ml 12 mol/L nitric acid was added into a three-necked flask equipped with a condenser and a thermometer and was reacted at 100°C for 8 h. After the reaction was completed, the oxidized activated carbon (denoted as AC-O) was prepared by washing with water and drying. Then 2 g AC-O was added into 50 ml of 0.1 mol/L NaOH aqueous solution, stirred for 12 h, filtered, washed and dried to obtain AC-ONa. 2 g AC-ONa was added into 20 ml halobenzene solution of a certain concentration, reacted at reflux temperature for a certain time, followed by being filtered, washed and dried to prepare halobenzene modified activated carbon (denoted as AC-Ph). 2 g of AC-Ph was added to 20 ml of concentrated sulfuric acid, and stirred at 150°C for 4 h. After the reaction was completed, AC-Ph-SO_3_H was prepared after being filtered and washed until neutral.

### 2.3 Structural Analysis of AC-Ph-SO_3_H

#### 2.3.1 Determination of Acid Functional Group Content

The acid contents of samples were analyzed using Boehm titration (Scheibe et al., 2010). The specific methods include: 1) Determination of strong acid content: 50 mg of sample was added into 20 ml 2 mol/L NaCl and ultrasonically treated for 30 min, followed with filtered and washed with water. Then 0.01 mol/L NaOH was used to titrate the filtrate with phenolphthalein as an indicator to determine the content of strong acids such as -SO_3_H; 2) Determination of the content of medium and strong acids: 50 mg of the sample was added into 20 ml of 0.01 mol/L NaHCO_3_ and ultrasonically treated for 30 min, followed with filtered and washed with water. Then the filtrate was titrated with 0.01 mol/L HCl with bromocresol green-methyl red as an indicator to determine the contents of strong acids such as -SO_3_H and medium-strong acids such as -COOH.

#### 2.3.2 Stability Test of Acidic Functional Group

The stability of -COOH and -SO_3_H in different solvents was tested as follows. 2 g carbon material and 20 ml different solvents were added to the reflux condensation device. After refluxing for a certain time, the content of acidic functional groups of the material was measured after being filtrated, washed with ethanol and distilled water and dried.

#### 2.3.3 Analysis of Surface Structure of AC-Ph-SO_3_H

The functional groups on the surface of the samples were analyzed by infrared spectroscopy. The used infrared spectrometer was Perkin Elmer 283 with DTGS/KBr detector. The test conditions were: the measurement wavenumber range was 4,000–400 cm^−1^, the resolution was 4 cm^−1^, and the acquisition rate was 20 times/min. The carbon skeleton of activated carbon before and after modification was tested by DXR laser microscope Raman spectrometer (laser wavelength 780 nm), the wavelength range was 50–3,250 cm^−1^, the exposure time was 5 s, the exposure times were 10 times, and the laser intensity was 5 mW.

### 2.4 Catalytic Performance of AC-Ph-SO_3_H in the Methylation of Palmitate

The catalytic performance of AC-Ph-SO_3_H in the preparation of biodiesel from methylation of palmitic acid was investigated. The conditions of reaction time, catalyst dosage and acid-alcohol molar ratio during the methylation reaction were studied. The typical catalytic process was as follows: 0.01 mol of palmitic acid, 0.4 mol of methanol and catalyst accounted for 2.5 wt% of palmitic acid was added into a three-necked flask equipped with a thermometer and a reflux condenser for 6 h at reflux temperature.

## 3 Results and Discussion

### 3.1 Preparation of AC-Ph-SO_3_H

The modification process of halogenated benzene coupled with -COOH mainly affected the content of sulfonatable groups on the surface of the materials, which had an important effect on the subsequent sulfonation process. Therefore, the effect of different halogenated benzenes (including iodized benzene, brominated benzene and chlorinated benzene) on the content of -COOH on AC-O surface at the reflux temperature of different solvents was investigated. In the catalytic process, the strong acid content in the acidic carbon-based solid acid will significantly affect the catalytic effect. Therefore, the influence of the concentration of halogenated benzene, the modification reaction time of halogenated benzene and the sulfonation temperature of concentrated sulfuric acid on the content of -SO_3_H during the preparation of AC-Ph-SO_3_H were investigated.

#### 3.1.1 Coupling Effect of Halogenated Benzene With -COOH in Different Solvents

The effects of halogenated benzenes (PhX) coupling with carboxyl groups on the surface of AC-O at reflux temperatures of different solvents (including cyclohexane, toluene, and DMSO) were investigated. The results were shown in Table 1. According to the results, the content of carboxyl groups on the surface of AC-O after oxidation of AC was 0.78 mmol/g. After modification by halogenated benzene in different solvents, the content of carboxyl groups on the surface decreased to a certain extent, indicating that the carboxyl groups on the surface of activated carbon was successfully coupled with halogenated benzene during the modification process. When cyclohexane was used as the solvent at 80°C of the reflux temperature, the -COOH content of AC-Ph was 0.62–0.65 mmol/g, which was only about 0.15 mmol/g lower than that of AC-O. When toluene being used as solvent at reflux temperature 110°C and DMSO being used as solvent at reflux temperature 180°C, the -COOH content on the surface of AC-Ph had little difference when modified with the same halogenated benzene. Under these conditions, the minimum content of carboxyl groups on the surface of the modified activated carbon was 0.28 mmol/g, indicating that the carboxyl groups being coupled were about 0.5 mmol/g and most of the carboxyl groups on AC-O had been replaced by benzene rings which can be easily sulfonated by concentrated sulfuric acid. When toluene was used as solvent, the -COOH content on AC-Ph modified by iodobenzene, bromobenzene and chlorobenzene were 0.28, 0.30, and 0.53 mmol/g, respectively. Since iodobenzene and bromobenzene have almost no difference in the modification of AC-O under the reflux condition of toluene, bromobenzene (PhBr) were used as the modification reagent due to its low economic cost and relatively stable performance, and toluene was used as the solvent when modifying AC-O in the follow-up studies. Under the selected conditions, the content of carboxyl groups on the surface of AC-Ph was 0.30 mmol/g, and the content of benzene rings grafted on the surface of activated carbon was about 0.50 mmol/g according to the difference in carboxyl content between AC-O and AC-Ph.

**TABLE 1:** Density of -COOH in AC-Ph modified with different halogenated benzene

Sample	PhX	solvent	Temp. (^o^C)	-COOH (mmol/g)
AC-O	/	/	/	0.78
AC-Ph	PhI	Cyclohexane	80	0.62
	PhI	Toluene	110	0.28
	PhI	DMSO	180	0.29
	PhBr	Cyclohexane	80	0.63
	PhBr	Toluene	110	0.30
	PhBr	DMSO	180	0.29
	PhCl	Cyclohexane	80	0.65
	PhCl	Toluene	110	0.53
	PhCl	DMSO	180	0.48

#### 3.1.2 Effect of Bromobenzene Concentration on -SO_3_H Content

**FIGURE 1:**
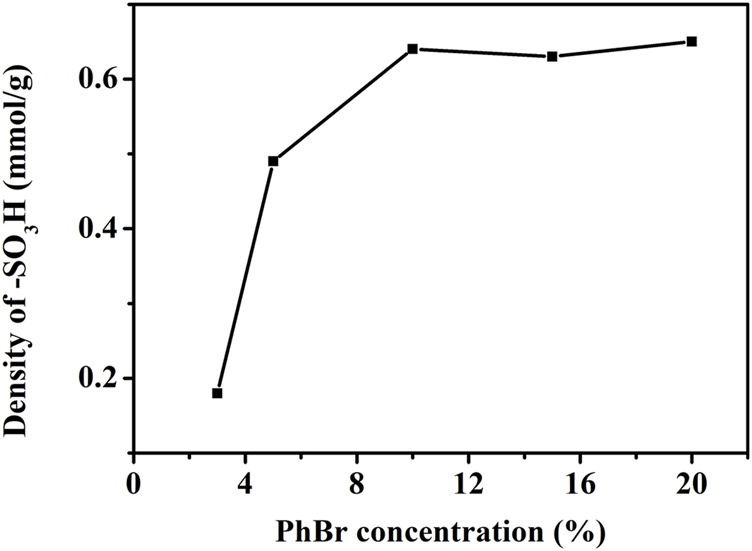
Density of -SO_3_H in AC-Ph-SO_3_H prepared under different PhBr concentration.

With bromobenzene as modification reagent and toluene as solvent, the effect of bromobenzene concentration on -SO_3_H content of AC-Ph-SO_3_H surface was studied. The results were shown in Figure 1. According to the results, as the concentration of bromobenzene increased, the content of -SO_3_H gradually increased. The concentration of bromobenzene showed a great influence on the -SO_3_H groups content on the surface of AC-Ph-SO_3_H. When the concentration of bromobenzene was 3%, the content of -SO_3_H was only 0.18 mmol/g. While when the concentration of bromobenzene was 10%, the content of -SO_3_H on the surface of AC-Ph-SO_3_H was 0.64 mmol/g. We speculated that the reason for this result was that the effective collision rate of bromobenzene with AC-O particles in solution was lower at a lower bromobenzene concentrations.

#### 3.1.3 Effect of Modification Time of Bromobenzene on -SO_3_H Content

**FIGURE 2:**
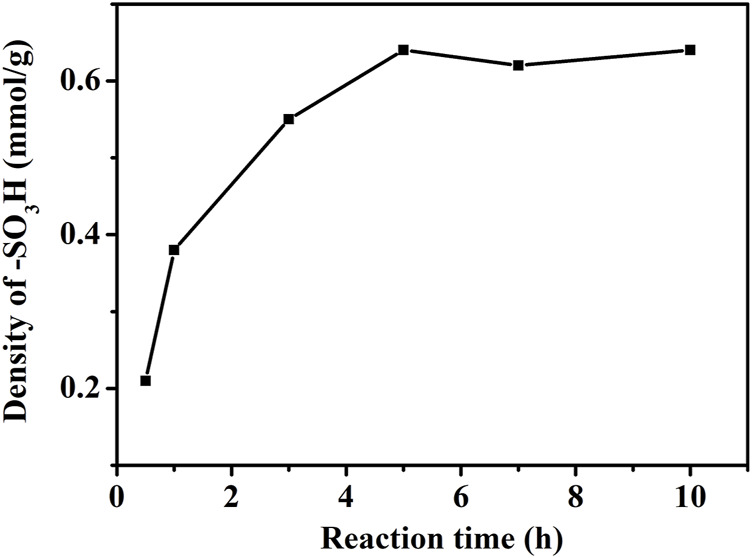
Density of -SO_3_H in AC-Ph-SO_3_H prepared under different PhBr treated time.

The effect of bromobenzene modification time on -SO_3_H content of AC-Ph-SO_3_H surface was shown in Figure 2. When the modification time was 0.5 h, the surface -SO_3_H content of AC-Ph-SO_3_H was 0.21 mmol/g and gradually increased with the prolongation of modification time. When the modification time reached 5 h, the content of -SO_3_H groups was 0.64 mmol/g and did not change when the time continued to prolong.

#### 3.1.4 Effect of Sulfonation Temperature on -SO_3_H Content

**FIGURE 3:**
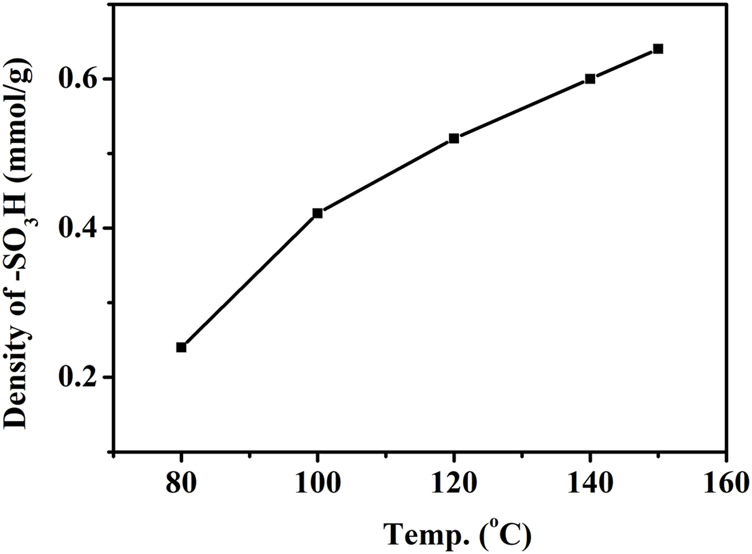
Density of -SO_3_H of AC-Ph-SO_3_H under different sulfonation temperature.

The effect of temperature on the -SO_3_H content of AC-Ph-SO_3_H during sulfonation with concentrated sulfuric acid was shown in Figure 3. When the temperature was low, the effect of sulfonation process was poor, and the content of -SO_3_H was only 0.24 mmol/g at 80°C. With the increasing of sulfonation temperature, the content of sulfonic acid groups on the surface of AC-Ph-SO_3_H gradually increased, which was 0.64 mmol/g when the temperature reached 150°C.

### 3.2 Structural Analysis

#### 3.2.1 Surface -COOH and -SO_3_H Contents of AC-Ph-SO_3_H and Its Precursors

The content of -SO_3_H and -COOH on AC-Ph-SO_3_H and its precursors in the preparation process were studied. At the same time, in order to verify the effect of bromobenzene modification on the sulfonated sites on the surface of activated carbon, the AC-SO_3_H and AC-O-SO_3_H prepared from the precursors AC and AC-O under the same sulfonation conditions were compared. The test results were shown in Table 2. According to Table 2, there was absolutely no -SO_3_H detected on the surface of AC-SO_3_H prepared by the direct sulfonation of AC. The reason was that there was little sulfonation site because of the fused ring structure on the surface of AC. The AC surface structure was damaged and resulted in a certain amount sulfonatable sites after oxidation with HNO_3_, which lead to generating a certain -SO_3_H group after sulfonation treatment of AC-O, but the content of -SO_3_H was only 0.15 mmol/g. While after being coupled with bromobenzene, the sulfonatable sites was increased on the surface of AC-Ph, so the amount of -SO_3_H on AC-Ph-SO_3_H surface could reach up to 0.64 mmol/g. According to the changes of -COOH content on the surface of the material, it can be seen that a large number of carboxyl groups was generated on the surface of AC during oxidation process; while the content of carboxyl groups decreased after the modification of bromobenzene, and a large number of sulfonic acid groups were generated after sulfonation of AC-Ph, indicating that bromobenzene was successfully combined with the surface carboxyl groups to introduce a large number of sulfonatable sites on the surface. The content of -COOH on the surface of AC-O was 0.78 mmol/g, while that of AC-O-SO_3_H obtained by treating AC-O with concentrated sulfuric acid was 0.69 mmol/g, which was slightly lower for the shedding of AC-O during the sulfonation process. After sulfonation of AC-Ph, the -COOH content on the surface of AC-Ph-SO_3_H was 0.54 mmol/g, which was higher than 0.30 mmol/g on the surface of AC-Ph. That was because concentrated sulfuric acid had a certain oxidation effect in the process of sulfonation which leaded to generate a certain amount of -COOH.

**TABLE 2:** Surface -COOH and -SO_3_H contents of AC-Ph-SO_3_H and its precursors

Sample	Density of -SO_3_H (mmol/g)	Density of -COOH (mmol/g)
AC	/	0.03
AC-O	/	0.78
AC-Ph	/	0.30
^a^AC-SO_3_H	0.03	0.28
^b^AC-O-SO_3_H	0.15	0.69
AC-Ph-SO_3_H	0.64	0.54

a Sulfonation of unoxidized AC.

b Sulfonation of AC-O, without bromobenzene treatment.

#### 3.2.2 Stability of AC-Ph-SO_3_H in Different Solvents

The stability of the prepared AC-Ph-SO_3_H was investigated at reflux temperatures of different polar solvents. The shedding of -SO_3_H groups under reflux treatment for 10 and 20 h was investigated respectively, and the results were shown in Table 3. As shown, the stability of the -SO_3_H group on the surface of AC-Ph-SO_3_H gradually decreased with the enhancement of solvent polarity. After refluxing in cyclohexane for 20 h, the -SO_3_H content of AC-Ph-SO_3_H decreased from 0.64 mmol/g to 0.58 mmol/g with a loss of only 9.4%; while refluxing in acetic acid for 20 h, the -SO_3_H content was only 0.25 mmol/g with the loss as high as 60.9%. The reason was that the -SO_3_H groups on the surface of the prepared materials were bonded to the surface aromatic rings in the form of C-S bonds, and when the polarity of the solution increased, the small molecular aromatic rings were more likely to fall off on the surface of the materials, resulting in a decrease in the content of -SO_3_H groups.

**TABLE 3:** Stability of AC-Ph-SO_3_H in different solvents

Solvent	Temp. (^o^C)	-SO_3_H (mmol/g)		Lost (%)	
		10 h	20 h	10 h	20 h
Non	/	0.64	0.64	/	/
Cyclohexane	81	0.61	0.58	4.7	9.4
Ethanol	78	0.54	0.50	15.6	21.9
Water	100	0.49	0.48	23.4	25.0
Acetic acid	118	0.30	0.25	53.1	60.9

Note: Reaction conditions: 2 g AC-Ph-SO_3_H, was added into 20 ml solvents and refluxed under refluxed temperature.

#### 3.2.3 Raman Spectroscopy

**FIGURE 4:**
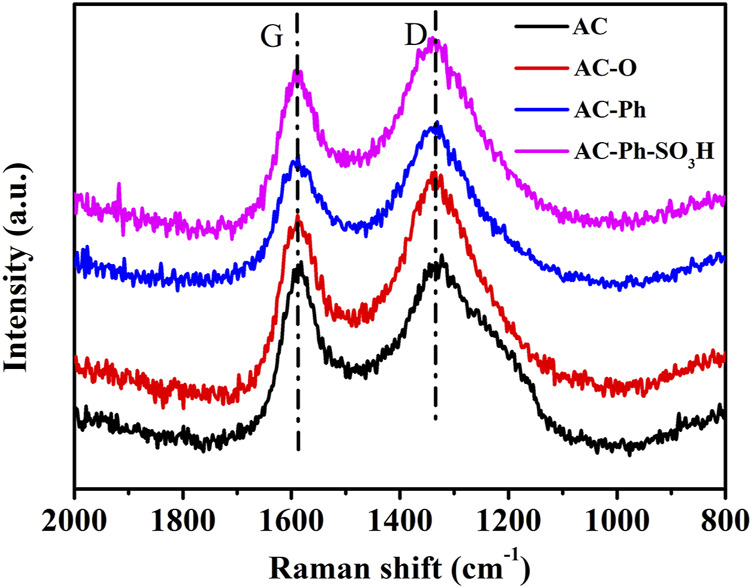
Raman spectrum of AC, AC-O, AC-Ph and AC-Ph-SO_3_H.

The Raman spectra of AC-Ph-SO_3_H and its precursors AC, AC-O, AC-Ph were characterized, and the carbon skeleton changes of activated carbon during oxidation, amination, and bromobenzene treatment were analyzed. The results were shown in Figure 4. According to Figure 4, there were mainly two broad peaks at 1,586 and 1,336 cm^−1^ in all carbon materials, of which the peak at 1,586 cm^−1^ was a G peak, representing the graphite structure of the carbon material framework; 1,336 cm ^−1^ was the D peak, which represented the non-graphitized amorphous structure. The intensity ratio I_D_/I_G_ of the D peak and the G peak can represent the graphitization degree of the carbon material. The smaller the I_D_/I_G_, the higher the graphitization degree of the material. The I_D_/I_G_ values of AC-Ph-SO_3_H and its precursors were shown in Table 4. According to Table 4, the I_D_/I_G_ value of AC was 1.029. After oxidation by nitric acid, the degree of graphitization of AC-O was reduced to 1.256, which indicated that the oxidation process had damaged the activated carbon structure to a certain extent, mainly by increasing the surface oxygen-containing functional groups. After further treatment with bromobenzene, the I_D_/I_G_ value of AC-Ph was 1.191, and its graphitization degree was improved to a certain extent. After AC-Ph was sulfonated with concentrated sulfuric acid, the I_D_/I_G_ value of AC-Ph-SO_3_H was 1.174, and its graphitization degree was slightly increased compared with AC-Ph, which was because of the shedding of some disordered small-molecule aromatic ring species.

**TABLE 4:** Raman spectrum I_D_/I_G_ of AC, AC-O, AC-Ph and AC-Ph-SO_3_H

Sample	AC	AC-O	AC-Ph	AC-Ph-SO_3_H
I_D_/I_G_	1.029	1.256	1.191	1.174

#### 3.2.4 FT-IR

**FIGURE 5:**
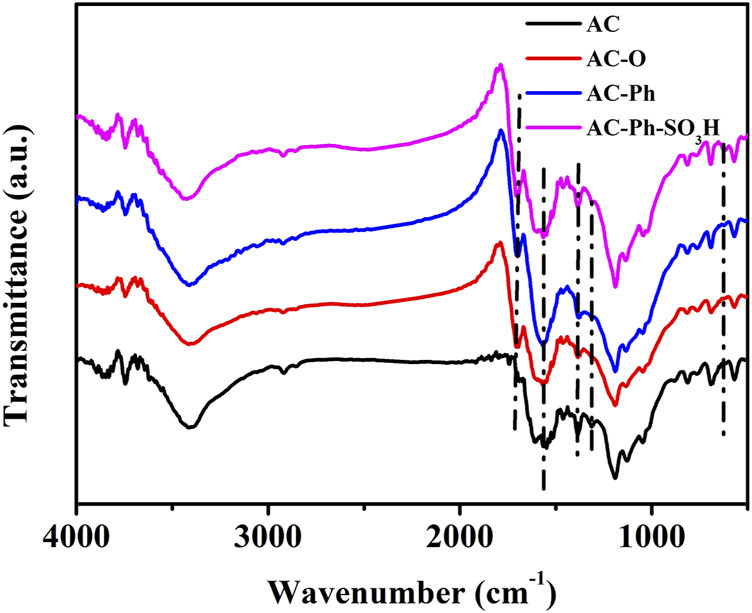
FT-IR spectrum of AC AC-O AC-Ph and AC-Ph-SO_3_H.

Functional groups on surface of AC-Ph-SO_3_H and its precursors AC, AC-O, AC-Ph were characterized by infrared spectroscopy, the results were shown in Figure 5. The strong absorption peak near 3,400 cm^−1^ was the O-H stretching vibration absorption peak. The absorption peak at 1,705 cm^−1^ was the stretching vibration of -COOH, and the absorption peak at 1,570 cm^−1^ was the stretching vibration of oxygen-containing functional groups such as quinoid structure. Except for AC, other samples all showed a certain absorption peak at 1,705 cm^−1^, indicating that the content of carboxyl groups on the surface of AC before treatment was very low, and a certain amount of carboxyl functional groups were added onto the surface of AC-O after oxidation. There was a broad absorption peak near 1,200 cm^−1^, which was the stretching vibration of functional groups containing oxygen atoms such as S = O, phenolic hydroxyl group, ether bond, lactones, etc. In AC-Ph-SO_3_H, in addition to the adsorption peak at 1,200 cm^−1^, there was also an absorption peak at 1,030 cm^−1^, both of which were S = O stretching vibration absorption peaks; an absorption peak was also found at 621 cm^−1^, which was the characteristic absorption peak of C-S, so it could be proved that a certain amount of -SO_3_H groups was generated on the surface of AC-Ph-SO_3_H after sulfonation.

#### 3.2.5 Specific Surface Area Analysis

**FIGURE 6:**
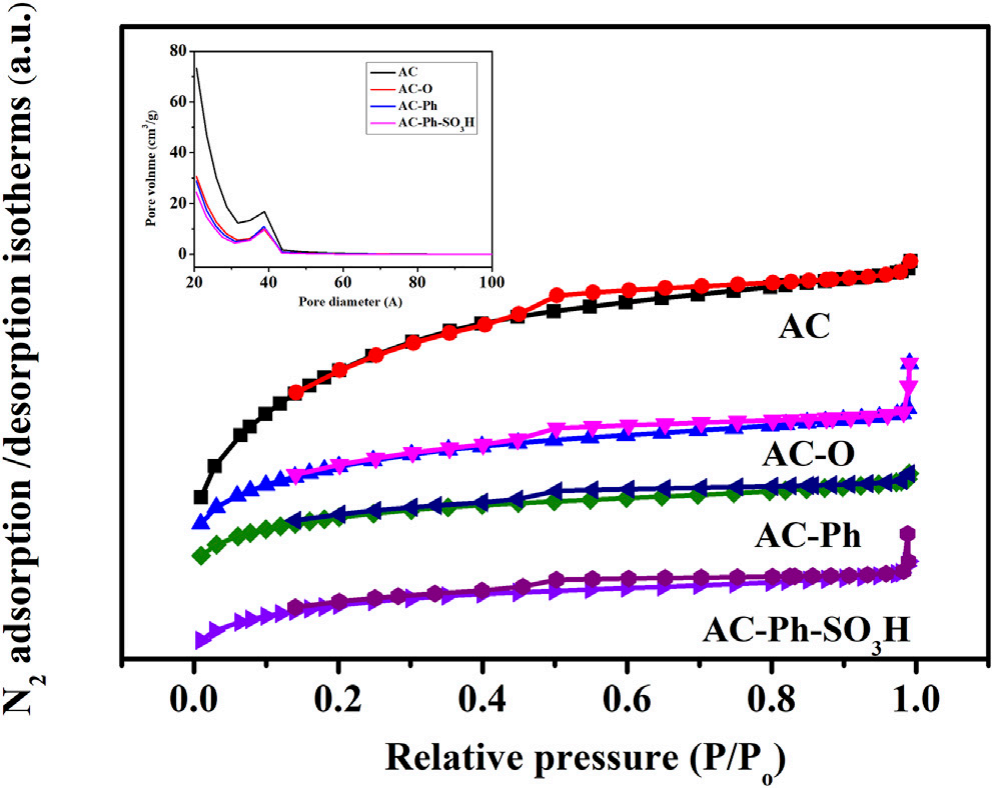
N_2_ adsorption‑desorption isotherms and pore size distribution for AC-Ph-SO_3_H and its precursors.

The N_2_ adsorption-desorption isotherms and pore size distribution of AC-Ph-SO_3_H and its precursors were shown in Figure 6, which were all belonged to type IV class and showed mesoporous structure indicating that the presence of a uniform mesoporous structure for AC-Ph-SO_3_H and its precursors. The surface areas were calculated with standard BET equation, which was shown in Table 5. According to Table 5, the BET surface area and pore volume changed greatly due to the destruction of the pore structure by the oxidation process, while the halogenated benzene modification and sulfonation process had relatively less effect on the pores. After modification, the obtained AC-Ph-SO_3_H still had a high specific surface area, of which was up to 472 m^2^/g, which provided the possibility of application in acidic catalytic systems combined with the high -SO_3_H density.

**TABLE 5:** BET surface area of AC-N of AC-Ph-SO_3_H and its precursors.

Samples	BET surface area (m^2^/g)	Pore volume (cm^3^/g)	Pore size (nm)
AC	1,286	0.77	2.4
AC-O	614	0.38	2.5
AC-Ph	528	0.31	2.5
AC-Ph-SO_3_H	472	0.30	2.5

### 3.3 Catalytic Performance of AC-Ph-SO_3_H in the Methylation of Palmitate Acid

#### 3.3.1 Effects of Reaction Conditions on Palmitic Acid Conversion and Methyl Palmitate Yield

**FIGURE 7:**
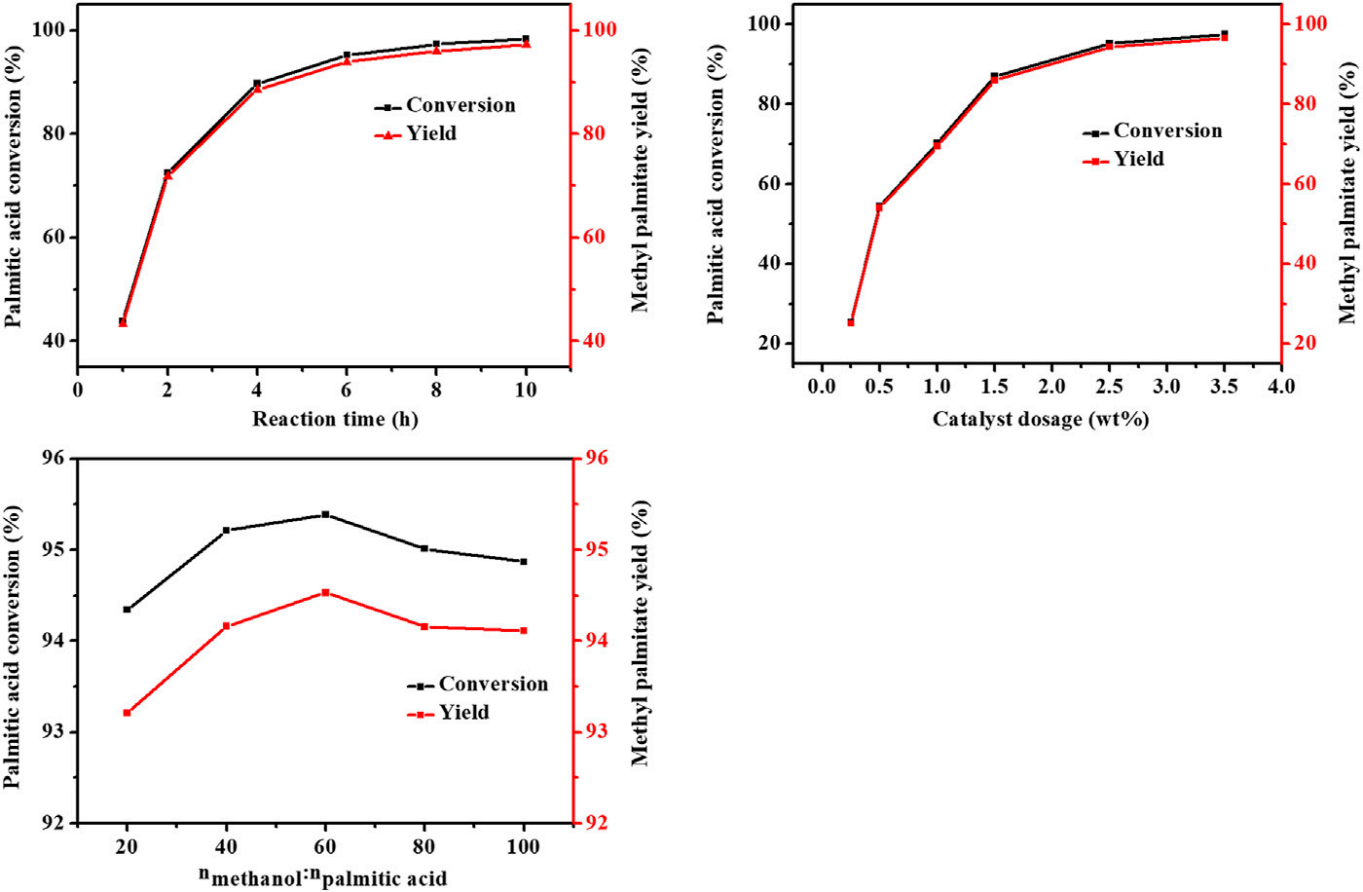
Effect of reaction conditions on methylation of palmitic acid.

The effects of reaction time, catalyst dosage and acid-alcohol molar ratio on the conversion of palmitic acid were investigated. The results were shown in Figure 7.

As shown in Figure 7, with the increase of the reaction time, the conversion rate of palmitic acid gradually increased. When the catalyst dosage was lower than 2.5 wt%, the conversion rate of palmitic acid increased significantly with the increase of the catalyst dosage. The conversion of palmitic acid was less affected by the molar ratio of methanol/palmitic acid and decreased to a certain extent when the amount of methanol was too large, which was because of the concentration of palmitic acid in the system diluted to a certain extent. The effect of reaction conditions on methyl palmitate yield was similar to the effect on conversion rate of palmitic acid, and the selectivity of methyl palmitate remained basically around 99% under almost all conditions. When the reaction time was 6 h, the catalyst dosage was 2.5 wt%, and the molar ratio of methanol/palmitic acid was 40, the palmitic acid conversion rate was 95.2% and the yield of methyl palmitate was 94.2%. When the reaction time continued to prolong and the catalyst dosage continues increased to 3.5 wt%, the conversion rate and methyl palmitate yield was still increased but not obvious. When the molar ratio was 20, the conversion of palmitic acid was 94.3%, which was similar to that of 40 m ratio. But when the methanol/palmitic acid molar ratio was lower than 20, the palmitic acid was not easily to dissolve completely, which affected the progress of the reaction.

#### 3.3.2 Catalytic Effects Comparation of AC-Ph-SO_3_H and Its Precursors

**FIGURE 8:**
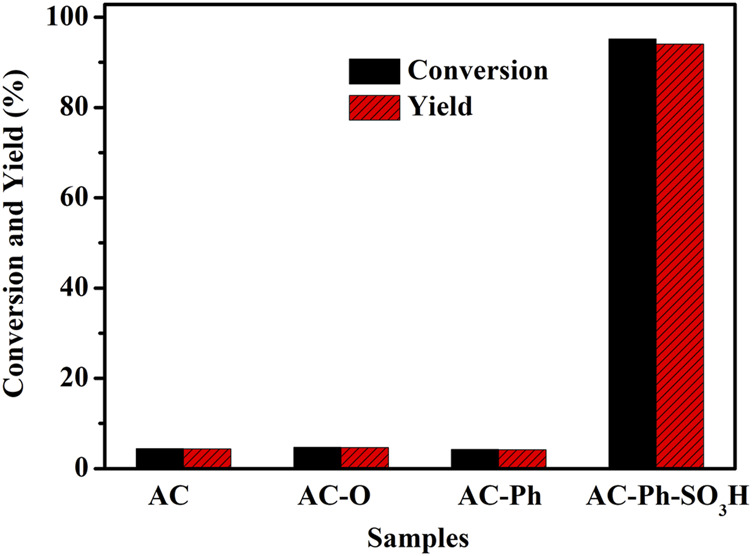
Conversion of palmitic acid catalyzed by AC-Ph-SO_3_H and its precursors.

In order to illustrate the modification effect more intuitively, the effects of AC, AC-O, AC-Ph, and AC-Ph-SO_3_H in catalyzing the reaction of methylation of palmitate were investigated respectively. The test results were shown in Figure 8. The reaction conditions were: 0.01 mol palmitic acid, 0.4 mol methanol, 0.39 g catalyst, and the reaction was carried out at reflux temperature (65°C) for 6 h. According to Figure 8, AC, AC-O, AC-Ph basically had no catalytic effect on palmitic acid methylation reaction, the conversion rates of palmitate acid were 4.4, 4.7, and 4.3%, respectively. While the conversion rate of palmitic acid under the catalysis of AC-Ph-SO_3_H was 95.2%, which indicated that the successful introduction of -SO_3_H on the surface of activated carbon played an important role in catalyzing the methylation of palmitate acid.

#### 3.3.3 Reusability of AC-Ph-SO_3_H in the Methylation of Palmitate Acid

**FIGURE 9:**
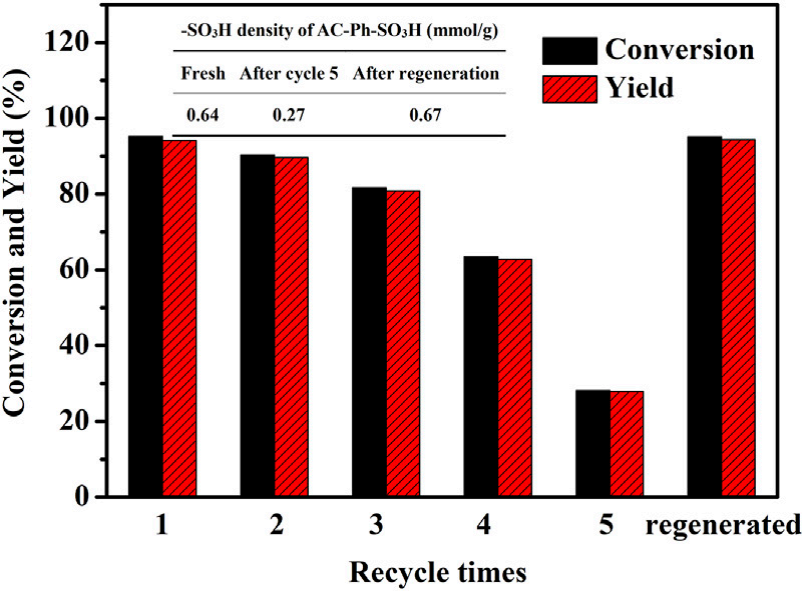
Recycle performance of AC-Ph-SO_3_H on methylation of palmitic acid.

The reusability of AC-Ph-SO_3_H in methylation of palmitic acid was investigated. In the study, the catalyst AC-Ph-SO_3_H was washed with methanol after use and fed under the same conditions. The test results were shown in Figure 9. The reaction conditions were: 0.01 mol palmitic acid, 0.4 mol methanol, 0.39 g catalyst, and reacted at reflux temperature (65°C) for 6 h. According to Figure 9, the conversion of palmitic acid was 95.2% at the first use of the catalyst. The conversion rate of palmitic acid was 90.4%, 81%, 7%, and 63.5% in the process of second to fourth reuse of the catalyst. In the fifth reuse, the conversion rate of palmitic acid was only 28.1%. The yield of methyl palmitate declined by a similar magnitude. The conversion rate and the yield gradually decreased with the increase of repeated times, but it showed good catalytic effect when it was reused three times. The reason for the gradual decrease of the catalytic effect was mainly including the formation of sulfonate and exfoliation of sulfonic acid groups on the surface of AC-Ph-SO_3_H during the catalytic process (Zhang et al., 2021), which had an important relationship with the presence of methanol and the polar environment of the reaction system.

After the catalyst was used for five times, the AC-Ph-SO_3_H was regenerated after being washed with methanol, dried, and sulfonated with concentrated sulfuric acid at 150°C for 4 h. As shown in Figure 9, the density of -SO_3_H on the surface of AC-Ph-SO_3_H was only 0.27 mmol/g after being used for five times, while reached 0.67 mmol/g after regeneration, which was even slightly higher than that of the fresh catalyst. At the same time, the conversion rate of palmitic acid also reached 95.1%. Which indicated that the structure of AC-Ph-SO_3_H was not destroyed in the catalysis process, although there were problems such as functional group shedding or masking.

#### 3.3.4 Comparison of Catalytic Performance With the Reported AC-Based Solid Acids

The catalytic performance of prepared AC-Ph-SO_3_H and the reported carbon-based sulfonic acid-type catalysts in the esterification of long-chain fatty acids were compared, and the results were shown in Table 6. As shown, most of the catalysts had good catalytic performance in the esterification reaction, but basically there was a problem that the catalytic efficiency decreased when they were recycled. The prepared AC-Ph-SO_3_H showed comparable catalytic performance to those reported catalyst, and showed advantages in terms of catalyst dosage and catalytic efficiency. Some of the catalysts (Niu et al., 2018) presented in the table were regenerated and showed excellent catalytic performance again. As shown in Entry 6, the conversion rate of fatty acid was only 28% when it was reused for the fifth time. After regeneration, the conversion rate of fatty acid reached 94% like that of catalyzed with fresh catalyst. At the same time, the density of -SO_3_H on the surface of the material also returned to the fresh level. Combined with these research results, carbon-based catalysts had great application potential in catalytic esterification to prepare biodiesel, while their stability and regeneration performance still need to be further explored.

**TABLE 6:** Comparison of catalytic performance with the reported carbon-based solid acids.

Entry	Solid acid catalyst	Density of -SO_3_H (mmol/g)	Catalyst amount (wt%)	Acid	Time (h)	Conv. in the first Cycle (%)	Reaction cycle	Conv. in the last cycle (%)	Ref.
1	AC-Ph-SO_3_H	0.64	2.5	Palmitate acid	6	95.2	3	81.7/N	This study
2	MLC-S	0.91	5	Oleic acid	6	92.3	5	72.9/N	Ma et al. (2020)
3	CS-SAC	1.48	5	Oleic acid	24	93	3	56/N	Bureros et al. (2019)
4	CS-SAC	0.85	7.5	Oleic acid	24	76	5	78/Y	Mendaros et al. (2020)
5	AC108	0.93	10	Oleic acid	6	89	5	89/N	Wang et al. (2014)
6	Heterogeneous acid catalyst synthesized from bamboo-AC	1.17	12	Oleic acid	3	96	5	28/N; 94/Y	Niu et al. (2018)
7	SO_3_H-KSC	—	2	palm fatty acid	1.5	98.7	6	60/N	Akinfalabi et al. (2019)
8	Bamboo-SO_3_H	—	4	palm fatty acid	1	95.8	4	71/N	Farabi et al. (2019)
9	HTC-S	—	3	palm fatty acid	2	92	3	82/N	Ibrahim et al. (2020)
10	ASHC-SO_3_H	1.4	10	Oleic acid	3	96.4	4	55.1/N; 95.4/Y	Cao et al. (2021)

Note: N represents no regeneration; Y represents regeneration.

## 4 Conclusion

Using activated carbon as raw material, AC-Ph-SO_3_H was prepared by oxidation with nitric acid, modification with halogenated benzene and sulfonation with concentrated sulfuric acid. With toluene as solvent and bromobenzene as modification reagent, high density of acidic group -SO_3_H were grafted on the surface of AC successfully. When the concentration of bromobenzene was 10%, the modification time of bromobenzene was 5 h, and the sulfonation temperature was 150°C, the surface -SO_3_H content of AC-Ph-SO_3_H was 0.64 mmol/g.

In the reaction of catalyzing the methylation of palmitate acid to prepare biodiesel, AC-Ph-SO_3_H showed a good catalytic performance. When the reaction time was 6 h, the amount of catalyst acid accounted for 2.5 wt% of palmitic acid, and the mole fraction of methanol/palmitic acid was 40, the conversion rate of palmitic acid was 95.2%. While the conversion rates of palmitate acid catalyzed by AC, AC-O, AC-Ph were only about 4.5%, which indicated the success of the modification process. In the study of the reusability of AC-Ph-SO_3_H as catalyst in methyl esterification of palmitate acid, the conversion rate of palmitate acid was still above 80% after three times of use, which indicated an excellent reusability.

Because unmodified activated carbon materials are difficult to be sulfonated to obtain activated carbon-based solid acid materials, the results of this study provided certain scientific support for the preparation of activated carbon-based solid acid materials. We believe that activated carbon materials with strongly acidic group have very good application prospects in the field of catalysis combined with its rich pore structure. However, like the reported amorphous carbon-based acid catalyst, the group of -SO_3_H on AC-Ph-SO_3_H was also easy to fall off after several reaction cycles. The specific reasons for -SO_3_H shedding and how to improve the stability of AC-Ph-SO_3_H need to be researched further. The application of AC-Ph-SO_3_H in the catalytic conversion of biomass also needs to be further developed.

## Data Availability

The original contributions presented in the study are included in the article/Supplementary Material, further inquiries can be directed to the corresponding authors.
